# Supplementary material for: Three Year Follow-Up of Reduced Dose of Direct Oral Anticoagulants for Extended Treatment of Venous Thromboembolism: An Ambispective Cohort Study
Source: Diagnostics (Basel). 2025 Sep 8;15(17):2283. doi: 10.3390/diagnostics15172283 (PMC12428014; doi:10.3390/diagnostics15172283)
Supplement: Supplementary file 1 [file diagnostics-15-02283-s001.zip › diagnostics-3806955-supplementary.pdf]

**Supplementary Table S1. Baseline characteristics sorting patients by the type of DOAC**

| | Overall 140 | Apixaban 103 | Dabigatran 8 | Edoxaban 9 | Rivaroxaban 20 | p |
|---|---|---|---|---|---|---|
| age (mean ±SD) | 71.72 (15.33) | 73.57 (14.16) | 61.43 (16.02) | 80.83 (11.86) | 62.75 (17.73) | 0.006 |
| Sex (male) (%) | 73 (52.1) | 50 (48.5) | 4 (50.0) | 8 (88.9) | 11 (55.0) | 0.140 |
| BMI (mean ±SD) | 27.45 (5.84) | 27.11 (6.00) | 30.45 (5.80) | 27.34 (NA) | 28.06 (5.16) | NA |
| smoking (%) | | | | | | 0.554 |
| No smoker (%) | 80 (70.8) | 56 (66.7) | 6 (85.7) | 8 (100.0) | 10 (71.4) | |
| Former smoker (%) | 32 (28.3) | 27 (32.1) | 1 (14.3) | 0 (0.0) | 4 (28.6) | |
| Active smoker (%) | 1 (0.9) | 1 (1.2) | 0 | 0 (0.0) | 0 | |
| Active cancer (%) | 18 (21.2) | 15 (23.8) | 0 | 3 (37.5) | 0 | 0.151 |
| Previous VTE (%) | 49 (35.5) | 37 (36.3) | 3 (37.5) | 1 (11.1) | 8 (42.1) | 0.433 |
| VTE family history (%) | 9 (7.4) | 5 (5.6) | 1 (16.7) | 0 | 3 (17.6) | 0.215 |
| Previous bleeding event (%) | 5 (3.6) | 2 (2.0) | 1 (12.5) | 1 (11.1) | 1 (5.6) | 0.239 |
| Site of VTE (%) | | | | | | 0.039 |
| PE | 43 (30.7) | 32 (31.1) | 4 (50.0) | 3 (33.3) | 4 (20.0) | |
| Cerebral vein thrombosis | 1 (0.7) | 0 | 1 (12.5) | 0 | 0 | |
| Isolated jugular vein thrombosis | 4 (2.9) | 3 (2.9) | 0 | 0 | 1 (5.0) | |
| Ovaric vein thrombosis | 1 (0.7) | 0 | 0 | 0 | 1 (5.0) | |
| Retinal vein occlusion | 1 (0.7) | 1 (1.0) | 0 | 0 | 0 | |
| SplVT | 2 (1.4) | 2 (1.9) | 0 | 0 | 0 | |
| LEDVT | 51 (36.4) | 38 (36.9) | 1 (12.5) | 1 (11.1) | 11 (55.0) | |
| LEDVT + PE | 27 (19.3) | 19 (18.4) | 1 (12.5) | 4 (44.4) | 3 (15.0) | |
| UEDVT | 5 (3.6) | 4 (3.9) | 1 (12.5) | 0 | 0 | |
| UEDVT + PE | 2 (1.4) | 2 (1.9) | 0 | 0 | 0 | |
| Other sites thrombosis | 3 (2.1) | 2 (1.9) | 0 | 1 (11.1) | 0 | |
| Risk factor (%) | | | | | | 0.186 |
| Persistent RF | 72 (51.4) | 55 (53.4) | 5 (62.5) | 3 (33.3) | 9 (45.0) | |

| | | | | | | |
|---|---|---|---|---|---|---|
| Transient RF | 12 (8.6) | 7 (6.8) | 0 | 3 (33.3) | 2 (10.0) | |
| Unprovoked | 56 (40.0) | 41 (39.8) | 3 (37.5) | 3 (33.3) | 9 (45.0) | |
| Causes (%) | | | | | | 0.658 |
| autoimmune disease | 13 (9.3) | 9 (8.7) | 1 (12.5) | 1 (11.1) | 2 (10.0) | |
| Active cancer | 21 (15.0) | 18 (17.5) | 1 (12.5) | 1 (11.1) | 1 (5.0) | |
| Liver cirrhosis | 1 (0.7) | 1 (1.0) | 0 | 0 | 0 | |
| Acute illness | 6 (4.4) | 3 (2.9) | 0 | 2 (22.2) | 1 (5.0) | |
| Multiple RF | 9 (6.4) | 8 (7.8) | 0 | 1 (11.1) | 0 | |
| Surgery | 4 (2.9) | 2 (1.9) | 0 | 1 (11.1) | 1 (5.0) | |
| Thrombophilia | 30 (21.4) | 21 (20.3) | 3 (37.5) | 0 | 6 (30.0) | |
| Leg injury | 1 (0.7) | 1 (1.0) | 0 | 0 | 0 | |
| Unprovoked | 55 (39.3) | 40 (38.8) | 3 (37.5) | 3 (33.3) | 9 (45.0) | |
| Concomitant therapies | | | | | | |
| antiplatelet (%) | 6 (4.3) | 3 (2.9) | 1 (12.5) | 1 (11.1) | 1 (5.0) | 0.418 |
| statin (%) | 39 (27.9) | 30 (29.1) | 4 (50.0) | 3 (33.3) | 2 (10.0) | 0.148 |
| Corticosteroids (%) | 19 (13.7) | 14 (13.6) | 1 (12.5) | 0 | 4 (21.1) | 0.510 |
| VTE recurrence (%) | 1 (0.7) | 1 (1.0) | 0 | 0 | 0 | 0.948 |
| Arterial events (%) | 4 (2.9) | 3 (2.9) | 1 (12.5) | 0 | 0 | 0.316 |
| Bleeding events (%) | | | | | | 0.461 |
| No bleeding event | 132 (94.3) | 96 (93.2) | 8 (100) | 8 (88.9) | 20 (100.0) | |
| CRNMB | 2 (1.4) | 2 (1.9) | 0 | 0 | 0 | |
| Major | 4 (2.9) | 4 (3.9) | 0 | 0 | 0 | |
| minor | 2 (1.4) | 1 (1.0) | 0 | 1 (11.1) | 0 | |

*BMI: body mass index VTE: venous thromboembolism, PE: pulmonary embolism, LEDVT: lower extremity deep vein thrombosis, UEDVT upper extremity deep vein thrombosis, SplVT: splanchnic venous thrombosis, CVC: central venous catheter, CRNMB: clinically relevant non major bleeding, RF: risk factor*
